# The Penicillin Pioneer: Alexander Fleming’s Journey to a Medical Breakthrough

**DOI:** 10.7759/cureus.65179

**Published:** 2024-07-23

**Authors:** Sumeet Chhabra, Avinash B Taksande, Pratiksha Munjewar

**Affiliations:** 1 Medicine, Jawaharlal Nehru Medical College, Datta Meghe Institute of Higher Education and Research, Wardha, IND; 2 Physiology, Jawaharlal Nehru Medical College, Datta Meghe Institute of Higher Education and Research, Wardha, IND; 3 Medical Surgical Nursing, Smt. Radhikabai Meghe Memorial College of Nursing, Datta Meghe Institute of Higher Education and Research, Wardha, IND

**Keywords:** bacterial infections treatment, howard florey, medical breakthroughs, antibiotic revolution, penicillin discovery, alexander fleming

## Abstract

Alexander Fleming’s discovery of penicillin is one of the most significant breakthroughs in medical history, revolutionizing the treatment of bacterial infections and saving countless lives. This report chronicles Fleming’s journey from his early life in rural Scotland to his pioneering work in bacteriology. It delves into his medical education and career, including his formative experiences during World War I that shaped his future research. The serendipitous discovery of penicillin in 1928, followed by the challenges of isolating and producing the antibiotic, is explored in detail. The report also highlights the crucial contributions of Howard Florey, Ernst Boris Chain, and Norman Heatley in developing penicillin into a widely usable therapeutic agent, particularly during World War II. Fleming’s achievements were recognized with the Nobel Prize in 1945 and numerous other honors in Physiology/Medicine. His personal life, continued research, and lasting impact on medicine are also discussed, emphasizing the enduring legacy of his work in the ongoing development of antibiotics and the transformation of medical practices. This comprehensive overview underscores the importance of curiosity, perseverance, and collaboration in scientific discovery, inspiring future researchers.

## Introduction and background

**Figure 1:**
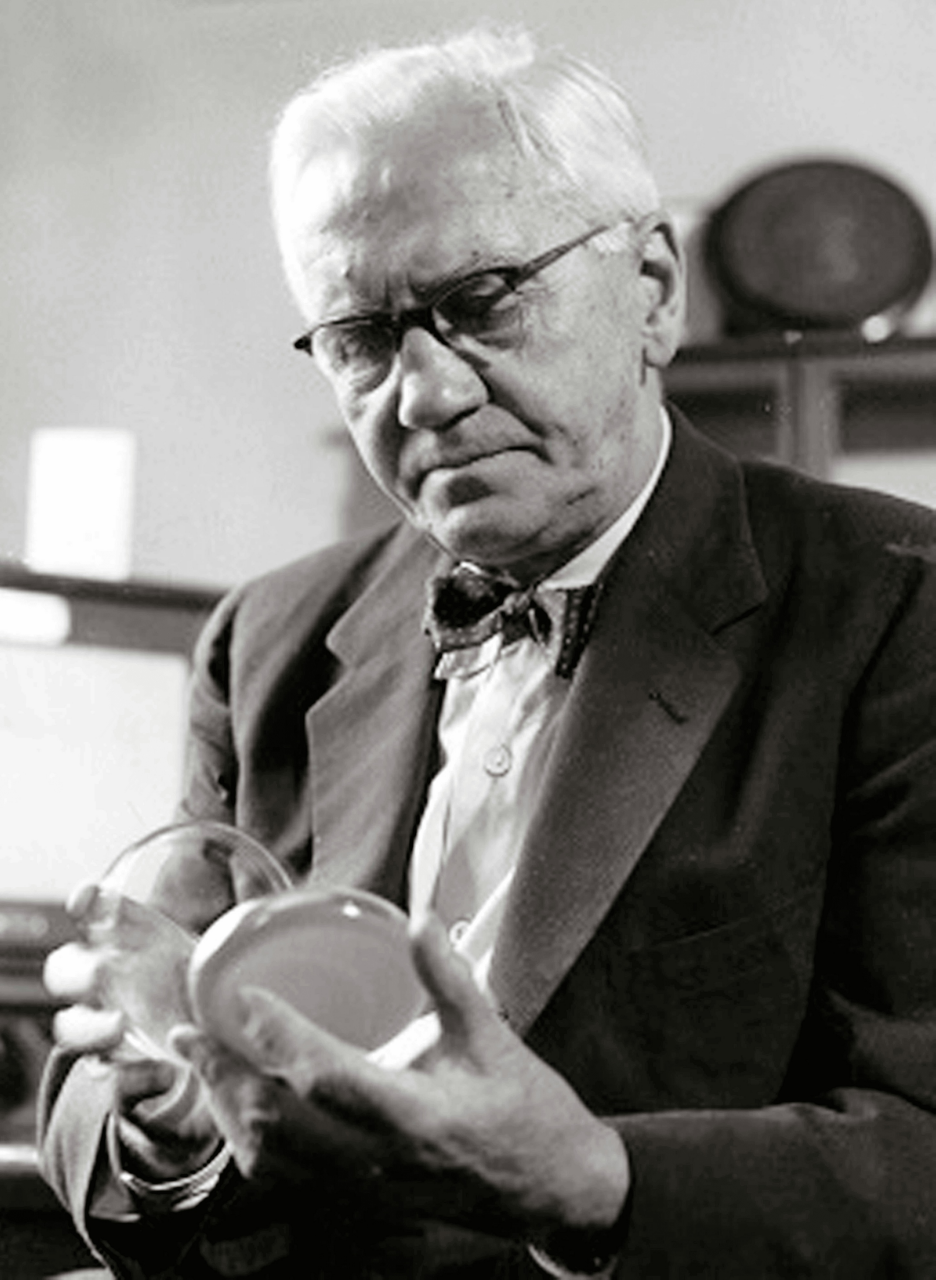
Alexander Fleming. Open-access journal under a CC-BY license Contributed by Imperial College Healthcare NHS Trust [3].

“In the realm of medical miracles, few discoveries rival the significance of penicillin in revolutionising the treatment of bacterial infections.” This quote encapsulates the profound impact of penicillin on modern medicine, highlighting its role in saving countless lives and transforming healthcare practices worldwide [1]. Alexander Fleming, a Scottish bacteriologist, is celebrated for his serendipitous discovery of penicillin in 1928 (Figure 1). His work marked a turning point in medical history, leading to the development of the first proper antibiotic. Before the advent of antibiotics, infant mortality, defined as the death of children before their first birthday, was approximately one in 20. Today, this rate has dramatically decreased to 3.5 deaths per 1,000 live births, a remarkable improvement largely attributed to the discovery of antibiotics [2]. Fleming’s discovery provided an effective treatment for bacterial infections and laid the groundwork for the antibiotic era, significantly reducing mortality rates and improving the quality of life for millions [2]. This report aims to provide a comprehensive overview of Alexander Fleming’s journey, from his early life and education to his medical career and the pivotal moment of discovering penicillin. It will also explore his challenges, penicillin’s development and widespread impact, and his lasting medical legacy. Through this examination, we can appreciate the significance of Fleming’s contributions and the enduring influence of his work on modern medical practices.

## Review

Early life and education

Alexander Fleming was born on August 6, 1881, in Lochfield, a rural area in Ayrshire, Scotland. He was the seventh of eight children born to Hugh Fleming, a farmer, and his second wife, Grace Stirling Morton. Growing up on a farm, young Alexander developed a keen sense of observation and curiosity about the natural world, which would later serve him well in his scientific endeavors [2,3]. Fleming’s early education occurred at local schools, where he demonstrated a natural aptitude for learning. Despite the limited resources available in the rural setting, he excelled in his studies, showing particular interest in science and nature. His academic potential was recognized early on, setting the stage for his future educational pursuits [2].

Medical education

Fleming’s academic excellence earned him a scholarship to attend Kilmarnock Academy, a significant step from his humble beginnings. This opportunity allowed him to receive a higher-quality education and further develop his academic interests, particularly in the sciences [4]. In 1901, Fleming moved to London to attend St. Mary’s Hospital Medical School. He initially intended to pursue a career in surgery. Still, his path took a decisive turn when he joined the research department under the mentorship of Sir Almroth Wright, a pioneer in immunology and vaccine therapy. Under Wright’s guidance, Fleming honed his skills in bacteriology and research, setting the foundation for his future groundbreaking work in antibiotics [5].

Medical career and influences

After completing his medical studies, Alexander Fleming joined the research department at St. Mary’s Hospital, working under the tutelage of Sir Almroth Wright. Wright was a leading figure in immunology and vaccine therapy, known for his innovative approaches to preventing and treating bacterial infections. Under Wright’s mentorship, Fleming developed a profound interest in bacteriology and conducted extensive research on the body’s immune responses to bacterial infections. This early career phase shaped his scientific approach and deepened his understanding of bacterial pathogens [6].

Experiences in World War I

Fleming served as a Royal Army Medical Corps captain during World War I. He was stationed in battlefield hospitals, where he witnessed firsthand the devastating impact of infected wounds on soldiers. These experiences were crucial in shaping his later research endeavors [7]. The horrific conditions of the trenches and the prevalence of septic wounds among injured soldiers made a lasting impression on Fleming. He observed that the antiseptics used at the time were often ineffective against deep-seated infections and could sometimes exacerbate tissue damage. These observations underscored the urgent need for more effective antibacterial treatments and fueled Fleming’s determination to find a solution. This period of his life profoundly influenced his future research, ultimately leading to his groundbreaking discovery of penicillin [8].

The discovery of penicillin

In September 1928, Alexander Fleming made one of the most fortuitous discoveries in medical history. Upon returning to his laboratory at St. Mary’s Hospital after a two-week vacation, Fleming noticed something unusual on a Petri dish that had been uncovered. The dish containing colonies of *Staphylococcus* bacteria had been contaminated by a mold, later identified as *Penicillium notatum*. Fleming observed that the mold had created a clear zone around itself where the bacteria could not grow [1]. Intrigued by this phenomenon, Fleming conducted experiments to understand the mold’s antibacterial properties. He found that the mold produced a substance that killed many harmful bacteria, including those responsible for diseases such as pneumonia, meningitis, and diphtheria. Fleming named this substance “penicillin” after the *Penicillium* mold. His initial observations and experiments demonstrated penicillin’s potential as a powerful antibacterial agent, marking the beginning of a new era in medical science [9].

Early challenges

Despite the promising results, Fleming faced significant challenges in isolating and producing penicillin in quantities sufficient for practical use. Extracting and purifying penicillin was complex and time-consuming, and the yields were initially meager. Fleming’s laboratory lacked the resources and expertise to overcome these technical obstacles [9]. Furthermore, the scientific community and pharmaceutical industry showed little interest in Fleming’s discovery. Many researchers were skeptical about the feasibility of producing penicillin on a large scale, and others were skeptical of its practical applications. This lack of support and funding hindered the progress of penicillin development, and for several years, Fleming’s groundbreaking discovery remained largely overlooked. Despite these early challenges, Fleming’s perseverance and the subsequent efforts of other scientists eventually led to the widespread adoption of penicillin, transforming it into a lifesaving antibiotic [10].

Development and impact of penicillin

The development of penicillin into a widely usable antibiotic was not the work of Fleming alone. In the late 1930s and early 1940s, a team of scientists at the University of Oxford, including Howard Florey, Ernst Boris Chain, and Norman Heatley, took on the challenge of furthering Fleming’s discovery. Florey and Chain recognized the potential of penicillin, and with Heatley’s expertise in biochemistry, they embarked on a project to isolate, purify, and produce penicillin in larger quantities [11]. The Oxford team made significant advancements in the production and purification of penicillin. They developed new methods for extracting and concentrating penicillin from mold cultures, significantly increasing yields. Their work included crucial experiments demonstrating the effectiveness of penicillin in treating bacterial infections in animals and humans. These advancements laid the groundwork for the mass production of penicillin, turning it from a laboratory curiosity into a practical and powerful therapeutic agent [12].

World War II and mass production

World War II provided the impetus needed to accelerate the development and production of penicillin. The high incidence of infected wounds and diseases among soldiers underscored the urgent need for effective antibacterial treatments. The success of penicillin in early clinical trials caught the attention of the United States and British governments, who recognized its potential to save countless lives [13]. With government backing, efforts to mass-produce penicillin were ramped up significantly. In the United States, pharmaceutical companies such as Pfizer, Merck, and Squibb developed new fermentation techniques to produce penicillin on an industrial scale. By the war’s end, penicillin was produced in large quantities, making it widely available for military and civilian use. Penicillin’s impact on medicine was profound and far-reaching. It drastically reduced the death rates from bacterial infections, such as pneumonia, rheumatic fever, and syphilis. Its success paved the way for discovering and developing other antibiotics, revolutionizing the treatment of bacterial infections and significantly improving public health outcomes. The era of antibiotics had begun, transforming medical practices and saving millions of lives worldwide [14].

Recognition and legacy

In 1945, Alexander Fleming, Howard Florey, and Ernst Boris Chain were awarded the Nobel Prize in Physiology or Medicine. This prestigious award recognized their contributions to the discovery and development of penicillin, which revolutionized the treatment of bacterial infections and saved countless lives during World War II and beyond [6]. In addition to the Nobel Prize, Fleming received numerous other accolades and honors for his groundbreaking work. He was knighted by King George VI in 1944, becoming Sir Alexander Fleming. His achievements were celebrated globally, and he received honorary degrees, memberships in prestigious scientific societies, and numerous awards from various countries and institutions, solidifying his legacy as a pioneering scientist [6].

Lasting impact on medicine

The discovery of penicillin paved the way for the development of a wide array of antibiotics, fundamentally transforming medicine. Penicillin’s success demonstrated the potential of natural compounds in fighting bacterial infections, leading to the discovery and development of many other antibiotics, such as streptomycin, tetracycline, and erythromycin. These antibiotics have been crucial in treating many bacterial infections, reducing mortality and morbidity worldwide [9]. The introduction of penicillin brought about significant changes in medical practices. It revolutionized the treatment of bacterial infections, making once life-threatening conditions easily treatable. Penicillin and other antibiotics drastically reduced the need for invasive surgical procedures to treat infections and enabled more effective management of infectious diseases. The widespread availability of antibiotics also improved surgical outcomes by preventing postoperative infections, thereby contributing to advancements in various medical fields [15]. Penicillin’s impact extended beyond individual patient care to public health at large. It played a crucial role in controlling epidemics and reducing the spread of infectious diseases, contributing to increased life expectancy and improved quality of life globally. Fleming’s discovery marked the beginning of the antibiotic era, fundamentally altering the landscape of medicine and establishing a legacy that continues to influence medical research and practices [16].

Personal life

In 1915, Alexander Fleming married Sarah Marion McElroy, an Irish nurse. Their marriage was marked by mutual support and shared interests in the medical field. They had one son, Robert Fleming, born in 1924. Robert followed in his father’s footsteps, pursuing a medical career and becoming a general practitioner. Alexander Fleming’s family life provided stability and support throughout his groundbreaking research and professional endeavors [2]. After the death of Sarah in 1949, Fleming remarried in 1953. His second wife was Dr. Amalia Koutsouri-Voureka, a Greek colleague and bacteriologist whom he had met during his work. A shared passion for science and mutual respect characterized their marriage. Dr. Koutsouri-Voureka continued to support and collaborate with Fleming in his later research activities [17].

Later years and death

Alexander Fleming continued researching and teaching at St. Mary’s Hospital Medical School in his later years. He remained active in the scientific community, sharing his knowledge and experiences with the next generation of scientists and medical professionals. His commitment to research and education persisted, contributing to advancements in bacteriology and antibiotic therapy [2]. Alexander Fleming passed away on March 11, 1955, from a heart attack. He was interred in St. Paul’s Cathedral in London, an honor befitting his monumental contributions to science and medicine. Fleming’s legacy continued to be celebrated posthumously through numerous honors and commemorations. His work remains a cornerstone of modern medicine, and he is remembered as one of the greatest scientists of the 20th century. Monuments, plaques, and institutions worldwide bear his name, ensuring his medical contributions are remembered and revered for generations [18].

## Conclusions

Alexander Fleming’s journey from a rural Scottish farm to the forefront of medical innovation is a testament to his relentless curiosity, perseverance, and dedication. His serendipitous discovery of penicillin in 1928 marked a turning point in medical history, laying the foundation for the antibiotic era and revolutionizing the treatment of bacterial infections. Despite initial challenges and skepticism from the scientific community, Fleming’s groundbreaking work, supported by the collaborative efforts of Howard Florey, Ernst Boris Chain, and Norman Heatley, led to the mass production of penicillin, saving countless lives during World War II and beyond. Fleming’s contributions earned him numerous accolades, including the Nobel Prize, and his legacy endures in the continued development of antibiotics and the transformation of medical practices. His personal life, marked by supportive relationships and ongoing research, further highlights his unwavering commitment to advancing science. Alexander Fleming’s story underscores the profound impact that one discovery can have on the world, inspiring future generations to pursue scientific inquiry with passion and tenacity.
